# Practices and challenges related to antibiotic use in paediatric treatment in hospitals and health centres in Niger and Uganda: a mixed methods study

**DOI:** 10.1186/s13756-023-01271-7

**Published:** 2023-07-11

**Authors:** Grace Mambula, Deborah Nanjebe, Aurelia Munene, Ousmane Guindo, Aichatou Salifou, Abdoul-Aziz Mamaty, Susan Rattigan, Sally Ellis, Nathalie Khavessian, Rob W van der Pluijm, Caroline Marquer, Irene Aicha Adehossi, Céline Langendorf

**Affiliations:** 1 Epicentre - Médecins Sans Frontières, 14-34 Avenue Jean Jaurès, Paris, 75019 France; 2 Epicentre Uganda, Kabale Road, MUST Campus, P.0. Box 1956, Mbarara, Uganda; 3 Epicentre Niger, Quartier Plateau, Boulevard Mali Béro, Issa Beri rue 31, Porte N° 93, Niamey, BP : 13330 Niger; 4 GARDP Foundation, Chemin Camille-Vidart 15, Geneva, 1202 Switzerland; 5 Hôpital National Niamey, BP 238, Niamey, Niger

**Keywords:** Antimicrobial resistance, Antibiotics, Paediatrics, Prescription, Sub-saharan Africa

## Abstract

**Background:**

Antibiotic resistance is a significant public health problem and is responsible for high mortality in children and new-borns. Strengthening the rational use of antibiotics and improving the quality and access to existing antibiotics are important factors in the fight against antibiotic resistance. This study aims to provide knowledge on the use of antibiotics in children in resource-limited countries in order to identify problems and possible avenues for improvement of antibiotics use.

**Methods:**

We conducted a retrospective study in July 2020 and collected quantitative clinical and therapeutic data on antibiotic prescriptions between January and December 2019 in 4 hospitals or health centres in both Uganda and Niger, respectively from January to December 2019. Semi-structured interviews and focus groups were conducted among healthcare personnel and carers for children under 17 years of age, respectively.

**Results:**

A total of 1,622 children in Uganda and 660 children in Niger (mean age of 3.9 years (SD 4.43)) who received at least one antibiotic were included in the study. In hospital settings, 98.4 to 100% of children prescribed at least one antibiotic received at least one injectable antibiotic. Most hospitalized children received more than one antibiotic in both Uganda (52.1%) and Niger (71.1%). According to the WHO-AWaRe index, the proportion of prescriptions of antibiotics belonging to the Watch category was 21.8% (432/1982) in Uganda and 32.0% (371/1158) in Niger. No antibiotics from the Reserve category were prescribed. Health care provider’s prescribing practices are rarely guided by microbiological analyses. Prescribers are faced with numerous constraints, such as lack of standard national guidelines, unavailability of essential antibiotics at the level of hospital pharmacies, the limited financial means of the families, and pressure to prescribe antibiotics from caregivers as well as from drug company representatives. The quality of some antibiotics provided by the National Medical Stores to the public and private hospitals has been questioned by some health professionals. Self-medication is a widespread practice for the antibiotic treatment of children for economic and access reasons.

**Conclusion:**

The study findings indicate that an intersection of policy, institutional norms and practices including individual caregiver or health provider factors, influence antibiotic prescription, administration and dispensing practices.

**Supplementary Information:**

The online version contains supplementary material available at 10.1186/s13756-023-01271-7.

## Background

Antimicrobial resistance (AMR) is a major threat to global health [1]. In the year 2019, an estimated 1.27 million deaths were attributable to AMR. Western sub-Saharan Africa was estimated to be the worst affected region, with 27.3 deaths per 100,000 persons attributable to AMR and 114.8 deaths per 100,000 persons associated with AMR [2]. The treatment of antimicrobial resistant infections is more costly due to the longer duration of illness, additional tests and the use of more expensive drugs [3]. Factors that can affect AMR prevalence are the rate of inappropriate prescription and dispensing of antimicrobials, inadequate diagnostic infrastructure, inappropriate access to effective health care and the absence or suboptimal enforcement of regulations with regards to, for instance drug availability and/or prescription practices [4–6]. Another clear risk factor for AMR is the widespread availability of substandard or falsified antibiotics [7]. Numerous initiatives aimed at improving antibiotic use have been taken. Importantly, the World Health Organisation (WHO), through the AWaRe (Access, Watch, Reserve) initiative, has classified antibiotics into three categories: the ‘Access’ category contains antibiotics with a narrow working spectrum and low potential of inducing AMR. Antibiotics in these categories are considered as appropriate empiric treatments for regularly encountered infections. The ‘Watch’ category contains antibiotics that should be used in specific circumstances, due to a wider mode of action and a higher risk of inducing resistance. Finally, the ‘Reserve’ category contains antibiotics that should only be used as a last resort, for instance in case of proven multidrug resistant infections [8]. The AWaRe classification serves as a tool to obtain insights, summarize and to compare in the use of antibiotics in different settings and countries [9, 10]. In addition, the WHO has set a target for 2023, by which at least 60% of prescribed antibiotics globally should be from the Access category, as a way of reducing the inappropriate use of antibiotics [11]. The AWaRe classification is now firmly integrated in the List of Essential Medicines List (EML) [12]. The use of the AWaRe classification in alignment with the EML aims to improve the empiric antibiotic prescription by providing guidance for common infections in adults and children. In order to reduce inappropriate antibiotic use, quantification of antibiotic use is necessary. In addition, factors influencing both healthcare seeking behaviour as well as healthcare providing behaviour should be identified and understood. This study aims to provide insights in the antibiotic prescription behaviour of health care providers in both Niger and Uganda. In order to provide insight into antibiotics use, the antibiotics administered are classified according to the WHO 2019 AWaRe classification [13]. In addition, we conducted a qualitative study aimed at identifying the factors contributing to antibiotic prescription as well as factors influencing the use or demand for antibiotics by the end user.

## Methods

### Study design

Retrospective quantitative data on the prescription of antibiotics were collected from 4 sites each in Uganda and Niger. The second part of the study was an exploratory qualitative study aimed at obtaining insights in the perspectives of stakeholders on the use of antibiotics, through semi-structured interviews (SSI) and focus group discussions (FGD).

### Study settings

In Uganda, 4 study sites were selected were in and around Mbarara, the main commercial and administrative centre of Mbarara District: Mbarara City Council Health Centre III (MCC), Kabwohe Health Centre IV (KHC), Mbarara Regional Referral Hospital (MRRH) and Holy Innocent Children’s Hospital (HICH). In Niger, study sites were selected in Niamey: Hôpital National Niamey (HNN), and around Maradi: Centre Hospitalier Regional de Maradi (CHR), Hôpital District Guidan-Roumdji (HD) and Centre de Santé Intégré de Madarounfa (CSI) (Supplementary Table 1).

See Supplementary Fig. 1a and 1b for an overview of the health care systems for Uganda and Niger respectively.

### Data collection

#### Collection of quantitative data

Quantitative data on antibiotic prescriptions were collected retrospectively on a systematic sampling of encounters over 1 year. An encounter was defined as a first-time consultation (and not follow-up) for an illness in out-patients and the first day of admission for in-patients. Only data from children < 17 years of age who attended a general out-patient clinic or were newly admitted in a general paediatric medical ward that occurred on the 1st and 15th days of the month from January to December 2019 (or next workday in case of holidays or weekends) were included. Of these encounters, for out-patients, we enrolled all patients seen for an initial consultation (and not follow-up) on the survey days who had at least one antibiotic prescribed while for in-patients we enrolled all patients, admitted on the survey days, that had at least one antibiotic prescribed as part of their treatment on admission and collected information on all antibiotics prescribed during the course of hospitalisation. The data were collected by consultation of hospital-based registers and clinical notes. Hospital-based registers are used in both countries to record the list of patients seen in an out-patient clinic or admitted in a ward daily (admission logs). They typically provide information on patient biodata, diagnoses, and in out-patients, treatments given. We used these hospital-based registers to select all the patients seen on the 1st and 15th days of the month. For out-patients, where the diagnoses or treatments were not provided, the biodata in the registers were used to trace the patients’ files to obtain the required treatment information for our study. In the case of in-patients, clinical notes filled by the medical staff in the patients’ files were used to obtain information on the antibiotics prescribed during hospitalisation.

For the enrolled patients, we recorded basic demographic data, diagnoses and antibiotics that were prescribed. These data were aggregated at the facility and then country level and the antibiotics prescribed were grouped by the WHO AWaRe classification list published in 2019 [13].

#### Collection of qualitative data

For the qualitative part of the study, we conducted SSIs with health care personnel who were employees of the health centre and were directly involved with patient care (doctors, nurses, pharmacists and drug dispensers). We invited caregivers older than 18 years of age who had brought the children to the study facility for medical consultation for the FGDs. One or two FGDs, comprised of 3 to 8 participants, were organized per health care facility. Both the semi-structured interviews and FGDs had a standardized interview guide which was used as a tool to guide the discussions and elicit responses. For each of the countries, interviews were conducted by an experienced qualitative researcher. The interviews and FGDs were audio-recorded for transcription after written and verbal informed consent were obtained and lasted about 45 minutes per participant/FGD session. FGDs were conducted in the local languages with a translator when necessary, depending on the needs of the interviewee (Hausa or Lingala in Niger and Luganda or Runyankore in Uganda).

### Data analysis

Each consultation encounter was anonymized by assigning a unique identifier number. Diagnoses were coded with MedDRA® version 22.0 English. Data collected were entered into REDCap by one member of Epicentre at the respective research centre. Databases were managed by the Epicentre Mbarara and Maradi data management teams in Uganda and Niger respectively. R^®^ version 4.0.2 was used to clean the data and carry out descriptive analyses. This analysis was performed for all study participants. The demographic distribution and proportion of patients that received antibiotics were described. For continuous variables, the mean and standard deviation were given. Categorical variables were described using percentages. Missing data were considered to be missing at random, and no imputation was applied.

Qualitative interviews were transcribed verbatim by the qualitative researchers. Data analysis process involved manually conducting a thematic analysis by the members of the research team. This included developing codes and themes, analysing themes for meanings, patterns, synergies and contradictions. Initially, 2 experienced investigators independently reviewed the transcripts and manually developed codes. These codes were discussed with the other members of the study team. Where disagreements arose about particular codes, these were settled by discussion with members of the study team to reach a consensus. As more codes emerged iteratively from the data, initial codes were re-named where appropriate and then all codes were further grouped into subcategories.

### Ethics approvals

The protocol, informed consent form and participant information sheet were approved by the Mbarara University of Science and Technology Research Ethics Committee (ref 24/07–19) and Uganda National Council for Science and Technology (ref HS 2708) in Uganda, and by the Comité National d’Ethique pour la Recherche en Santé du Ministère de la Santé Publique, de la Population et des Affaires Sociales du Niger (n° 043/2018) in Niger. Written and verbal versions of the Participant Information and Informed Consent were presented to the participants in the local language, English or in French by suitably qualified and experienced study investigators.

## Results

### Quantitative study results

In Uganda antibiotics were prescribed in 47.6% (1839/3865) of the encounters (Supplementary Table 2). We collected details on antibiotic prescriptions and diagnoses for 1622 of these patients. We note that among in-patients in Uganda, the proportion of encounters with an antibiotic prescription was between 22.9 and 48.3% with the highest proportion seen in the private hospital. Among out-patients, on the other hand, this ranged between 29.8 and 65.7% with KHC, the level IV health centre, recording the highest proportion. In Niger, data was collected on 660 patients that received at least one dose of antibiotics. Due to the retrospective nature of the study no data on the number of patients seen in total in Niger is available, which prevents us from calculating an antibiotic prescription rate in Niger.

From this point, the results are exclusively for children enrolled who received antibiotics. On average, higher number of antibiotics were prescribed per hospitalized child (1.6 in Uganda and 1.9 in Niger) compared to number of antibiotics prescribed per child managed as an out-patient (1.1 in Uganda and 1.2 in Niger) (Table 1). For hospitalized children, in both countries, the vast majority of hospitalized children received at least one injectable antibiotic (98.5% in Uganda and 97.7% in Niger) and the mean duration of intra-venous antibiotics was similar between both countries (3.6 days (SD 2.6) in Uganda and 3.8 days (SD 2.0) in Niger). The proportion of hospitalized children receiving more than one antibiotic during the course of treatment, 53.9% in Uganda and 71.1% in Niger, was higher than in out-patients (14.9% in Uganda and 23.6% in Niger).

**Table 1:** Description of participants that received at least one antibiotic and antibiotics prescription by study site and by out-patients /In-patients, Uganda and Niger

	Uganda		Niger	
	In-patients	Out-patients	In-patients	Out-patients
Total participants (n)	267	1355	478	182
Female, n (%)	140 (52.4)	653 (48.2)	207 (43.3)	79 (43.4)
**Age of participants**				
Mean age, years (SD)	1.8 (2.9)	4.8 (4.5)	3.3 (3.1)	4.1 (4.5)
Total < 5 years, n (%)	231 (86.5)	845 (62.4)	365 (76.4)	136 (74.7)
Total ≥ 5 years, n (%)	36 (13.5)	510 (37.6)	113 (23.6)	46 (25.3)
Mean age children < 5 years, years (SD)	0.8 (1.0)	1.9 (1.2)	1.9 (1.3)	1.9 (1.2)
Mean age children ≥ 5 years, years (SD)	8.2 (2.8)	9.6 (3.8)	7.8 (2.9)	10.8 (3.8)
**Antibiotic prescriptions**				
Total antibiotics treatment (n)	432	1578	932	226
Total injectable antibiotics (n)	415	67	773	15
Children with at least one injectable %	263 (98.5)	59 (4.4)	467 (97.7)	15 (8.2)
Mean duration, days (SD)	3.6 (2.6)	5.9 (3.0)	3.9 (1.9)	4.8 (1.2)
Injectable	3.6 (2.6)	2.1 (2.3)	3.8 (2.0)	1 (0)
Oral	4.0 (3.0)	5.9 (2.6)	4.8 (0.9)	5.1 (0.6)
Children with multiple different antibiotics^1^ (%)	144 (53.9)	202 (14.9)	340 (71.1)	43 (23.6)
Average of antibiotics prescribed per child	1.6	1.1	1.9	1.2
Children with combination antibiotics^2^ (%)	130 (48.7)	127 (9.4)	249 (52.1)	31 (17.0)

^1^ Multiple antibiotics: More than one antibiotic prescribed during treatment. This includes changes of antibiotic regimens during the course of treatment.

^2^ Combination antibiotics: More than one antibiotic prescribed at the same time e.g., Ampicillin + Gentamicin.

In Uganda, among in-patients, the most common antibiotic prescribed was ampicillin (16%) while amoxicillin-clavulanic acid (28.1%) was the most prescribed among out-patients. In Niger, on the other hand, the combination of ceftriaxone + gentamicin was the most prescribed among in-patients (21.2%) while amoxicillin (49.1%) was the most prescribed antibiotic among out-patients. Details of antibiotic prescriptions divided by hospital and setting (in-patient and out-patient care) are provided in supplementary tables 3a and 3b.

Using the 2019 AWaRe Classification of Antibiotics from WHO, the proportion of prescriptions of antibiotics belonging to the Access and Watch categories were 78.2% (1550/1982) and 21.8% (432/1982) in Uganda and 68.0% (787/1158) and 32.0% (371/1158) in Niger respectively. The Watch category antibiotics were mainly prescribed in in-patients in Niger, whereas they were mainly prescribed in out-patients in Uganda due to frequent use of cefixime in the private health structure. No antibiotics from the Reserve category were prescribed in either country (Table 2).

**Table 2:** AWaRe Classification of Antibiotics prescribed in the study sites in Uganda and Niger

Category	Antibiotic	Listed in 8th EMLc 2019	Uganda *n*, (%)				Niger *n*, (%)		
			Total N = 1982	In-patients	Out-patients	Total N = 1158	In-patients	Out-patients	
Access	Total		1550 (78.2)	373 (18.8%)	1177 (59.4%)	787 (68.0)	564 (48.7%)		223 (19.3%)
	Amoxicillin	yes	365	3	362	210	84		126
	Amoxiclav	yes	445	2	443	32	32		0
	Ampicillin	yes	232	144	88	148	132		16
	Benz. benzylpenicillin	yes	3	9	3	0	0		0
	Benzylpenicillin	yes	12	12	9	0	0		0
	Cefalexine	yes	5	9	5	0	0		0
	Cloxacillin	yes	109	21	88	21	8		13
	Cotrimoxazole	yes	19	19	0	24	0		24
	Doxycycline	yes	8	9	8	0	0		0
	Flucloxacillin	yes	54	2	52	0	0		0
	Gentamicin	yes	119	102	17	282	282		0
	Metronidazole	yes	81	10	71	53	23		30
	Nitrofurantoin	yes	3	9	3	0	0		0
	Phenoxymethylpenicillin	yes	6	1	5	1	1		0
	Procaine-benzylpen.	yes	69	57	12	0	0		0
	Tetracycline	yes	20	0	20	16	2		14
**Watch**	**Total**		**432 (21.8%)**	**78 (3.9%)**	**354 (17.9%)**	**371 (32.0%)**	**368 (31.8%)**		**3 (0.2%)**
	Azithromycin	yes	24	3	21	0	0		0
	Cefixime	yes	193	0	193	13	13		0
	Cefotaxime	yes	2	1	1	8	8		0
	Ceftriaxone	yes	112	68	44	331	331		0
	Ciprofloxacin	yes	43	5	38	11	11		0
	Clarithromycin	yes	8	0	8	0	0		0
	Erythromycin	yes	12	0	12	7	4		3
	Levofloxacin	yes	2	0	2	0	0		0
	Meropenem	yes	0	0	0	1	1		0
	Neomycin	no	35	1	34	0	0		0
	Ofloxacin	yes	1	0	1	0	0		0
**Reserve**	**Total**		**0**	**0**	**0**	**0**	**0**		**0**

EMLc = Essentiel Medicines List for children

In Uganda, among in-patients, the most common diagnoses recorded were sepsis (22.9%) and respiratory tract infection (15.1%) while among out-patients respiratory tract infection (29.9%) and bacterial infection (13.7%) were the most common. In Niger, pyrexia (19.5%) and malaria (14.0%) were the most common diagnoses among in-patients while pyrexia (27.0%) and cough (17.6%) were the most common among out-patients.

### Qualitative study results

#### Participants’ characteristics

In Uganda, 18 health care workers participated in semi-structured interviews and 24 care-givers participated in focus group discussions. In Niger, 19 health care workers participated in semi-structured interviews, and 10 health care workers and 30 caregivers participated in focus group discussions (Table 3).

**Table 3:** Categorization of participants to semi-structured interviews (SSI) and focus group discussions (FGD) in Uganda and Niger

Country	Category	Number	SSI/FGD
Uganda	Medical doctor	4	SSI
	Medical/Clinical officer	5	SSI
	Nursing staff	5	SSI
	Pharmacy staff	4	SSI
	Caregivers	24	FGD
Niger	Medical doctor	8	SSI
	Nursing staff	12	SSI
	Nursing staff	9	FGD
	Caregivers	30	FGD

### Results

The main themes that emerged through the semi-structured interviews and the focus group discussions are discussed below. Each theme discussed is illustrated by some quotes.

#### Regulations concerning access to and prescription of antibiotics

Across both countries, antibiotics can be accessed with relative ease. Health care workers and caregivers pointed to poor drug regulations. There is the belief that giving caregivers access to unregulated private pharmacies or road-side market shops exposes patients to poor quality antibiotics. *“(The private sector) needs to be regulated by the national council and by the districts. There is an inspector, but he is not qualified, it leaves a gap in regulation. There are guidelines for all health facility levels but there is no enforcement and there is no budget.” (Doctor, MRRH, Uganda).* A phenomenon that was unique to Uganda was the role medical representatives play in antibiotic prescription practices at the health facilities. They visit health facilities often and contact prescribers directly to inform them of the latest drugs or formulations in the market including antibiotics, bypassing the health facility administration and National Medical Stores (NMS). Health providers who prescribe these branded drugs are incentivised by medical representatives: *“The brands are brought by medical representatives who represent various drug companies. They educate us on what is in the market because no one educates us. [Although] They are not authorized to come to the clinician, […] they come directly to the clinical officer not to the hospital. […]. In return it is scratch my back I scratch yours, you receive a ‘cup of tea’ like 5 000–10 000 UGs, or samples that you can stock in your personal facilities like 3–4 but mostly it is mainly cash. […].” (Clinical Officer, MRRH, Uganda)*.

#### Knowledge gaps among health care providers

In both Uganda and Niger, various health providers at the facilities acknowledged that there are knowledge gaps in the use of antibiotics and recommended Continuous Medical Education (CME) for staff as well as peer learning to improve the rational use of antibiotics.

*“Above all, we need training and continuous training, to insist on retraining, and to sensitize health care workers to the dangers of antibiotics.“ (Doctor, CHR Maradi, Niger).*

In Uganda, the most referenced guidelines were the Uganda Clinical Guideline and the WHO Integrated Management of Childhood illness (IMCI). Health care workers reported consulting several other guidelines to inform their antibiotic prescription choices, while taking into consideration factors like clinical presentation, availability and affordability of drugs, personal preference based on expertise, ease of guideline access and quality of content as well as previous experience in managing a particular condition. *“I use Medscape and UpToDate App online. It’s for health workers and students. Uganda Clinical Guidelines are really basic- meaning it is over simplified, especially when you are in place like a referral hospital.” (Medical Officer, MRRH, Uganda) “We have treatment guidelines in each room, but they are hard to follow. We need a simple book for children. I use the Basic Paediatric Protocol and I also use the Uganda Clinical Guideline 2016.” (Clinical Officer, MRRH, Uganda). “Since we are under hospital management, we follow [WHO] IMCI. We have to read more and consult wider literature”. (Clinical Officer, HICH, Uganda).* In Niger on the other hand, there is limited knowledge of a national antibiotics protocol or standardized clinical guideline that health care workers can refer to.

#### Poor stock management and budgetary constraints

Common to both countries is free health care services to children under the age of 5 years entailing free consultations and free access to drugs and other services. However, in all the facilities in this study, drugs, including antibiotics and other consumables are not always available. *“Free access to drugs is not 100% possible because sometimes, products are out of stock, even ceftri [ceftriaxone], and we have to write prescriptions, but all tests and hospitalization are free of charge.“ (Nurse, HN Niamey, Niger).*

The challenges faced with the non-availability of antibiotics in the facilities were related to limited fixed budgets that do not match increasing consumption levels at the health facility, high antibiotic prescription rates and inadequate stock management. *“Yes, we have free access, since we order at the district level depending on benefits. It does not cover the whole month, so afterwards, we write a prescription.“ (Nurse, CSI Madarounfa, Niger).*

Unavailability of antibiotics at the health facilities has significant implication on the choice prescribed, duration, formulation, course of treatment, and adherence. It also disconnects prescribing and dispensing processes which leads to incomplete antibiotic treatment courses, non-adherence, exposure to poor-quality antibiotics and delays in commencing treatment with sometimes fatal outcomes. *“Some health providers write the change in prescription, some don’t write, others tell patient to buy or take alternative. Or we prescribe for the days available then change to another antibiotic that is available.” (Doctor, MRRH, Uganda). “[…] because the product is not there, you have to buy it outside. Sometimes, the caregiver has to take a taxi, and by the time they bring the product back, the child may have died.“ (Doctor, HN Niamey, Niger).*

*“[...] Some mothers can’t pay, you can give her a prescription, she will keep it up to three days without buying the products. [...] She will tell you that she doesn’t even have enough money to buy food. This is our biggest problem; you start antibiotic therapy after 2 days, and you stop.“ (Doctor, HN Niamey, Niger).*

#### Non-availability and high cost of microbiological investigations

Among health care workers, doctors are aware that a rational prescription of antibiotics should be informed by bacteriological analysis. However, because of the high cost or lack of availability of these tests in the facilities, this resource is not routinely used; the cost of a microbial test is higher than the cost of antibiotics. In Uganda, MRRH was the only facility that had culture and sensitivity tests offered to patients for free, whereas it is available at a cost in other facilities. In addition, delays in receiving culture results of between 5 and 14 days, high cost and lack of testing supplies and unavailability of culture testing over the weekend limit the use of microbiological services, which in turn results in increased empirical prescription practices. *“We use this antibiotic dual therapy [ceftriaxone-gentamicin] to increase synergistic action. Drugs are more effective when used concomitantly. Sometimes we do not even have a diagnosis. Those treatments are given blindly, as we say.“ (Doctor, CHR Maradi, Niger).*

#### Staff shortages

High workload was reported as a key issue for antibiotics adherence instructions. Most health care workers could not give caregivers as much information on drug use in the required depth and quality.

*“Look at the formulation, and the workload. I see too many children and only spend 10 minutes with them not 30–40 minutes. So, I cannot give them all the needed information.” (Clinical Officer, MRRH, Uganda)*

In Niger, it was noted that antibiotics given several times per day are difficult to administer due to staff shortage. This led doctors to prescribe ceftriaxone with or without gentamicin more often than ampicillin. When ampicillin is given, the dose is reduced from the recommended 3 doses per day to 2 doses per day.

*“We start with injections of ampicillin. Before, we used to give 3 doses per day. We reduced the treatment to 2 doses per day because it is difficult to give 3 doses per day, considering the lack of personnel. [] We haven’t increased the dose, we divide the total dose given over 24 hours into 2 doses instead of 3.“ (Nurse, HN Niamey, Niger)*

In Niger, because of staff shortages, there is not a uniform availability of trained doctors in some facilities, resulting in nurses prescribing antibiotics. *“Our problem is that nurses prescribe antibiotics extensively, in almost all cases, they systematically give antibiotics, when we see the cases, we rectify this during medical rounds.“ (Doctor, CHR Maradi, Niger)*.

### Challenges with paediatric antibiotic formulations

Health care workers and Caregivers complained of struggling to give oral antibiotics to younger children because some are too bitter or too sweet. For older children, the pill burden is sometimes considered as high. *“There is when the tablet is bitter and when the child tastes its bitterness and you bring it back tomorrow, the child cries and refuses to take it.” (Caregiver, Kabwohe HC, Uganda)* In Uganda, health providers report numerous challenges with intravenous (IV) formulations due to inadequate skills on IV administration. For example, difficulties in cannula insertion and retention, high number of children on IV forms with fewer staff to administer the medications, lack of water for injection and the fact that injections are painful for children. In Niger, on the other hand, parenteral routes are more preferred and considered as the most effective and appropriate way to ensure good adherence to treatment by medical staff and caregivers. Indeed, several health care professionals consider that caregivers (mainly mothers) do not adhere to oral drug dosage at home, which is also acknowledged by some of the caregivers interviewed.

*“For mothers, you know the mentality of people from Niger, as long as they don’t get injections, they have received no treatment, but when they get injections, they think it is the right treatment.“ (Doctor, HN Niamey, Niger)*

Some health care workers pointed to the challenge of having several types of syrup formulations for the same drug produced by different manufacturers which can be confusing to caregivers especially when they don’t have clear instructions on how to reconstitute and administer these drugs. For parenteral formulations, many antibiotics are not adapted to paediatric use leading to a lot of wastage. *“Where do you store diluted antibiotics like one gram vial and you need 250 mg only, where do you keep the 750 mg?” (Pharmacist, MCC, Uganda)*.

### Knowledge of antibiotics and self-prescription among caregivers

In both Uganda and Niger, irrespective of the facility where the interviews took place and patient type, there was an awareness of specific antibiotic names but not the word “antibiotic” itself among caregivers. The ones most known were amoxicillin, penicillin and metronidazole.

*“Like you go to the hospital, and they tell you your child is suffering from an infection, and they don’t tell you the meaning of the infection. Whenever I take the child to the hospital, they tell me infection and they give me antibiotics. I really don’t understand antibiotics. All I know is that the doctors tell you we are going to give you antibiotics, but I don’t know what they are.” (Caregiver, MRRH, Uganda)*

In both Uganda and Niger, the self-prescription of antibiotics is commonly reported in a cross-section of care givers who access both private and public health facilities. This practice entails the caregiver prescribing for their child the antibiotics the child has used previously if symptoms are the same, relying on a friend’s advice whose child had similar symptoms or having a diagnosis made on telephone from a health provider with whom they are friends with. Caregivers mentioned high costs of seeking care as one of the reasons for self-prescribing. *“I bought from the clinic (private, often unregulated). There is one time when I came here (health centre), they gave me Amoxyl [amoxicillin] for children and my child got healed then second time I bought Amoxyl from the clinic.” (Caregiver, Kabwohe HC, Uganda) “I buy amoxi [amoxicillin] if my child has a cold, and if he has diarrhoea, I give him cipro [ciprofloxacin].” (Caregiver, CHR Maradi, Niger)*.

#### High indirect cost of antibiotic treatments

Although treatment at public health facilities is free, caregivers must contend with high indirect costs associated with treatment. For out-patient care, daily administrations of injectable antibiotics result in high transportation costs. For both out- as well as in-patients, loss of income for the caregiver are seen as important limiting factors to treatment adherence. *“I do not stay with my child; he is under my mother’s care. Just brought him when the condition worsened and since I brought him, I stopped working until my child gets well so am at home with him all the time.” (Caregiver, MCC, Uganda)*.

#### Suggestions for improvement by health care workers and caregivers

Both health care workers as well as caregivers made suggestions aimed at improving the prescription, use of antibiotics, and the availability of antibiotics. Interestingly, both parties mentioned the need for regulations of the availability of antibiotics to be enforced by the government. Health care workers in Uganda suggested stronger regulations aimed at reducing the influence of pharmaceutical representatives. In Uganda and Niger health care workers felt a strong need for ongoing training in antibiotic prescription practices, as well as the need for uniformity of either national or local guidelines. *“We should have antibiotics therapy committees in every hospital, even in every ward, then set up protocols for each pathology in each ward, or publicize national protocols at all levels, carry on with the training of health care workers so that there is enough medical staff and so that we can do high-quality work.” (Doctor, CHR Marad, Niger)*.

It is felt that investments should be made in recruitment and training of medical staff as staff shortages lead to high work pressure, suboptimal care and errors in drug administration. Health care providers think that the availability of microbiological tests such as blood cultures would reduce the extensive use or inform empirically chosen antibiotics. Both health care workers and caregivers suggested that investments should be made to improve the availability of commonly used antibiotics, as stock shortages are common and lead to self-medicating and a thriving poorly regulated private pharmaceutical market. On the other hand, the stock shortages can lead to patients not receiving full treatment courses or the use of the incorrect treatment regimens which are not suitable for specific treatments. *“Availability will improve prescription. We know the common OPD conditions in Uganda are pharyngitis, otitis and pneumonia. Let’s have drugs available for these conditions.” (Medical Officer, MRRH, Uganda)*.

## Discussion

This study describes the antibiotic prescription practices by health care providers in 4 health care centres in Uganda and 4 health care centres in Niger. Most antibiotics used fall within the WHO defined Access category. However, in the public reference hospitals Watch injectable antibiotics accounted for one-third to two-thirds of all antibiotics prescribed in Uganda and Niger respectively, while peripheral facilities prescribed oral antibiotics from the Access category for most patients. Reserve category antibiotics were neither prescribed nor available in the studied health care centres due to absence of this category in the national Essential Medicines Lists and high price if available in the private sector that limits their accessibility. A rapid increase in the consumption of Watch group antibiotics between 2000 and 2015 has been described, particularly in low- and middle-income countries (an increase of 165% versus 28% in high-income countries) [9]. However, in this study, Klein et al. recognize that some low-income regions, such as sub-Saharan Africa, are under-represented due to a lack of data. It is essential that surveillance of antibiotic consumption be expanded and systematized in resource-limited countries to monitor trends and assess the impact of measures taken to promote rational use of antibiotics and fight against antibiotic resistance.

In Uganda, amoxicillin was the most common antibiotic prescribed for out-patients in the lower level III and IV public facilities and we found a similar distribution in Niger where amoxicillin was the most prescribed in the two lower primary health care centres. The prominence of amoxicillin, drawing from the qualitative findings in Uganda, is linked to the introduction of the amoxicillin dispersible tablet which is the preferred formulation supplied by National Medical Stores (NMS) to both public and private non-profit health facilities in comparison to syrups. Another study conducted in Mbarara District among level III and IV health facilities showed that oral cotrimoxazole and amoxicillin were the most prescribed at these two levels [14]. In addition, we observed that the private hospital overwhelmingly had the highest number of encounters where antibiotics were prescribed in combination (i.e., more than one antibiotic prescribed at the same time). These findings are similar to other studies in the African continent, Malaysia and Pakistan, where private hospitals had higher polypharmacy rates than government hospitals [15–17].

The focus group discussions and interviews with caregivers and health care workers, respectively, provided important insights in the factors influencing antibiotic prescriptions and use. Among the health care workers, most treatments in Uganda’s and Niger’s health care centres occurred on an empiric basis due to the lack of microbiological investigations and antibacterial resistance surveillance in the respective settings. This results in health care providers prescribing broad spectrum antibiotics at the initiation of treatment or adding antibiotics during treatment when the patients fail to improve clinically. In addition, it prevents health care providers from narrowing the therapeutic spectrum of the prescribed antibiotics. In Niger, we note that the most common diagnosis recorded was that of pyrexia among both in- and out-patients and cough was the second common diagnosis among out-patients. In the lower primary care facilities (CSI and HD) especially, nurses are mostly the ones that see patients and prescribe medications. As their training does not entail making a diagnosis and prescribing medications, this may explain the higher tendency of making less specific symptomatic (instead of syndromic) diagnoses. This may lead to misclassifying illnesses and could further influence the use of empirical broader spectrum antibiotics.

One remarkable outcome was the fact that antibiotic dosing schedules and regimens are adjusted due to the shortage of staff. We found that there was a fair number of prescriptions for combinations of ceftriaxone and gentamicin because it was said to be convenient (once a day administration) and there was the belief that there was good synergy between the two drugs. Furthermore, high workload was reported as a major complaint by health care workers in both countries which affects the amount and quality of information provided to caregivers about antibiotic use. The interviewed health workers mentioned that they were unable to give instructions on the reconstitution of syrups, specific treatment timings, treatment completion, side effects and how to manage them, avoiding sharing antibiotics and drug interactions. In qualitative research by Médecins Sans Frontières on its programs in Sudan, the Central African Republic, the Democratic Republic of Congo, and Guinea-Bissau,[18] a major reason for incomplete treatment was that many patients did not understand the prescriptions or were unable to remember the instructions given by the prescriber.

Another important factor is the financial status of the caregivers. Some doctors adapt their prescriptions according to the family’s means such as prescribing oral antibiotics instead of injectables or prescribing less expensive but less suitable antibiotics. Families sometimes cannot afford to buy the entire treatment regimen so the health care worker is forced to reduce the duration of treatment. In addition, stock-outs of certain treatments in pharmacies in healthcare facilities sometimes prevent patients from receiving an ideal treatment in terms of dose and duration. This situation often forces caregivers to take these prescriptions and buy antibiotics outside the health facilities, in private pharmacies or on the informal market via street vendors. Treatment is then dependent on the caregivers’ financial means and the quality of the antibiotics they buy.

From the focus group discussions, it is clear that caregivers are used to self-medicating for the treatment of a febrile illness. Closely linked to self-prescription is self-diagnosis, where previous experiences of the caregiver and those of their friends with a particular illness, is relied upon to “diagnose” their child’s illness and ultimately lead to self-prescription. These findings are similar to other LMICs in Africa and Asia where patients and caregivers described convenience, time and cheaper costs as being the main reasons why patients and caregivers self-prescribe on antibiotics [19, 20].

In both Niger and Uganda, regulations are in place to discourage the use of antibiotics without a prescription. In Niger, pharmacovigilance activities are coordinated by the Ministère de la Santé Publique, de la Population et des Affaires Sociales (Ministry of Public Health, Population and Social Affaires) guided by national laws to limit the dispensing of medications without a prescription [21]. Whereas in Uganda, The National Drug Authority is responsible for the access and use of antimicrobials under the guidance of the National Drug Policy and Authority Act as well as other national guidelines [22, 23]. However, we note that there is poor adherence to these regulations as caregivers are able to purchase antibiotics without prescriptions with relative ease outside of the hospital pharmacies. This poor adherence to regulations by those that sell antibiotics outside the hospital, coupled with the caregivers’ ability to purchase only the quantity of antibiotics they can afford are important factors that can drive antimicrobial resistance. In a study carried out in Mbarara, Uganda and Tanzania to study the antibiotic prescribing practices during the COVID-19 epidemic, almost 90% of mystery clients who came without a prescription were dispensed pharmaceutical products in Mbarara and a majority of these were antibiotics [24]. In addition, concerns about the quality of the available antibiotics by both health care personnel and caregivers were raised. Counterfeit medicinal products is a recognised global problem and in Africa, it is estimated that up to 50% of drugs are counterfeit in some settings [25, 26]. This too can create opportunities for antimicrobial resistance and can have a negative effect on treatment outcomes and overall health care costs [27].

In our study, we note that in Uganda, the work of health care workers is influenced by pharmaceutical representatives who stimulate the use of specific antibiotics. Some health care workers acknowledged that they rely on medical representatives as an important source of information about antibiotics. Furthermore, the pharmaceutical representatives encourage physicians to prescribe their branded drugs and reward them with incentives. They are seen to influence negatively antibiotic prescribing practices by encouraging practitioners to deviate from the treatment guidelines and prescribe more expensive, non-generic antibiotics. Studies have shown that the collection of incentives or gifts from pharmaceutical industry representatives lead to irrational prescriptions and a higher prescription rate of non-generic formulations [28, 29]. As the non-generic medications are typically more expensive, this will have an impact on the access to antibiotics especially for caregivers that are already in precarious financial situations.

Both caregivers and health care providers in this study highlighted opportunities of improving the prescription and use of antibiotics. In terms of drug formulations, suggestions have been made to introduce new formulations or adapt existing ones such as having once-daily treatments, high quality antibiotic syrups that are already reconstituted, dispersible tablets for under and over five-year-olds, access to paediatric antibiotic injectable doses to minimize errors, wastage and storage challenges. Health care providers in both countries expressed the need for additional and ongoing education on the topic of the treatment of infections. In addition, there is a clear need for uniform, easily accessible and comprehensive treatment guidelines. Compared to no intervention, health care professionals having access to CMEs improves their practice and to a lesser degree, improve patient outcomes [30, 31]. Attention however must be paid to the type of CME offered as not all approaches will have the same impact on behavioural change. A Cochrane review found that educational meetings have a greater impact on improving compliance with desired practice than other types of interventions such as text messages and using a multi-strategy approach might influence positively the effectiveness of these meetings [30]. Intensifying ongoing capacity building support for public and private health providers across the various health facility levels and delivered by qualified trainers will help in bridging some of the knowledge and practice gaps among health care providers. Caregivers have limited knowledge on infections, antibiotic use and types. It is crucial that contextualized awareness on antibiotics at individual, institutional and community levels be put in place to address these gaps.

### Limitations

This study has several limitations. We had some missing or incomplete data due to retrospective collection of the quantitative data. Missing data included no clear diagnoses at admission or discharge and duration of treatment. Data on the duration of hospital stay for each patient was not collected so we could not contextualize especially the duration of IV antibiotic use with duration of hospitalisation. This study only looked at what types of antibiotics were prescribed globally and did not make linkages between treatments given and diagnoses. Numerous patients registered suffered from several infections, and because of the use of multiple antibiotics within one patient, we were not able to assess whether the antibiotics were prescribed in accordance with local treatment guidelines or were in adherence with the WHO list of essential medicines recommendations which was out of the scope for this study. Finally, to a large degree, we were unable to estimate the consumption of antibiotics in the facilities due to unavailable data at the pharmacy level in the surveyed facilities.

## Conclusion

This study aimed to explore the practices and challenges related to antibiotic use in the treatment of paediatric patients in hospitals and health centres in Niger and Uganda. It was found that there are many factors that influence the prescription of antibiotics including poor antibiotic regulations, unavailability of adequate quantities of antibiotic, lack of microbiological investigations in health facilities, knowledge gaps among healthcare providers and staff shortages. Caregivers found the indirect costs of antibiotics high and often resort to self-medication. Investments should be made in recruitment and ongoing training of health care workers, while also ensuring availability of antibiotics, simple comprehensive guidelines and access to high quality and timely microbiological laboratory analyses. In addition, regulation of antibiotic market, both formal and informal as well as community awareness campaigns might reduce self-medicating behaviour. Investments aimed at preventing the misuse of antibiotics should be an intersection of policy, institutional norms and practices including individual caregiver and health provider factors. A multipronged approach is needed to tackle these intersecting factors while involving different players in the use of antibiotics.

## Electronic supplementary material

Below is the link to the electronic supplementary material.

Supplementary Material 1

## Data Availability

The data generated during this study are not publicly available to protect the privacy of the interview participants. They can however be made available upon reasonable request to the corresponding author.

## List of abbreviations

AMR: Antimicrobial Resistance
AWaRe: Access, Watch, Reserve
CHR: Centre Hospitalier Régional
CME: Continuous Medical Education
CSI: Centre de Santé Intégré
EML: Essential Medicines List
FGD: Focus Group Discussion
HD: Hôpital de District
HICH: Holy Innocent Children’s Hospital
HNN: Hôpital National Niamey
KHC: Kabwohe Health Centre
MCC: Mbarara City Council Health Centre
MedDRA: Medical Dictionary for Regulatory Activities
MRRH: Mbarara Regional Referral Hospital
NMS: National Medical Store
SOC: System Organ Class
SSI: Semi-structured Interviews
WHO: World Health Organisation
