# Supplementary material for: Practices and challenges related to antibiotic use in paediatric treatment in hospitals and health centres in Niger and Uganda: a mixed methods study
Source: Antimicrob Resist Infect Control. 2023 Jul 11;12:67. doi: 10.1186/s13756-023-01271-7 (PMC10337096; doi:10.1186/s13756-023-01271-7)
Supplement: Supplementary file 1 — Supplementary Material 1 [file 13756_2023_1271_MOESM1_ESM.docx]

***Supplementary Table 1:*** *Description of the types of facilities included in the study in Uganda and Niger*

| Type of Facility | Services |
| --- | --- |
| *Uganda* |  |
| Mbarara Regional Referral Hospital (MRRH) | Provides referral services and supportive supervision to the district level hospitals within its health zone. It  Specializes in medical and surgical care, basic research, and training of nurses and paramedical officers. |
| Kabwohe Health Centre IV (KHC) | In-patient care with wards for men, women, and children. It has an operating theatre, laboratory, blood transfusion service. It is a referral facility for 20-30 HC level II and III health facilities. |
| Mbarara City Council Health Centre III (MCC) | It has basic laboratory services, maternity and in-patient care. It is staffed by nurse aides, qualified nurses and clinical officers (physician assistants) |
| Holy Innocent Children’s Hospital (HICH) [Private Non-Profit] | Provides a wide range of specialist paediatric care for both in- and out-patients. Autonomous but supervised by and reports to the district health services. |
| *Niger* |  |
| Hôpital National Niamey (HNN)  [National Hospital] | It is the largest hospital in Niger and provides a wide range of specialized medical and surgical care. |
| Centre Hospitalier Régional de Maradi (CHR) [Regional Hospital Centre] | A regional referral hospital which receives patients from other centres that do not have the technical facilities or the necessary skills. It has medico-technical services (laboratory, medical imaging, pharmacy) and provide specialist medical and surgical services. |
| Hôpital du District Guidan-Roumdji (HD) [District Hospital] | Caters for a population of about 500,000. It treats referrals from its corresponding Integrated Health Centres. |
| Centre de Santé Intégré de Madarounfa (CSI) [Integrated Health Centre] | Integrated primary health care centre that provides only out-patient services. |

***Supplementary Figure* *1a:*** *Healthcare structure in Uganda*

**
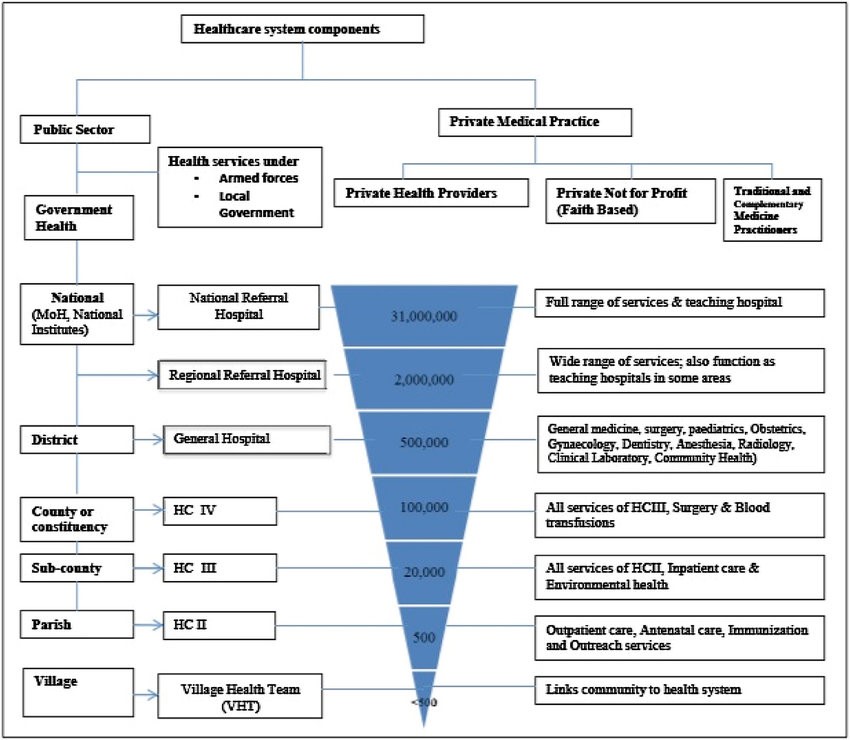
**

***Supplementary Figure* *1b:*** *Healthcare structure in Niger*

**
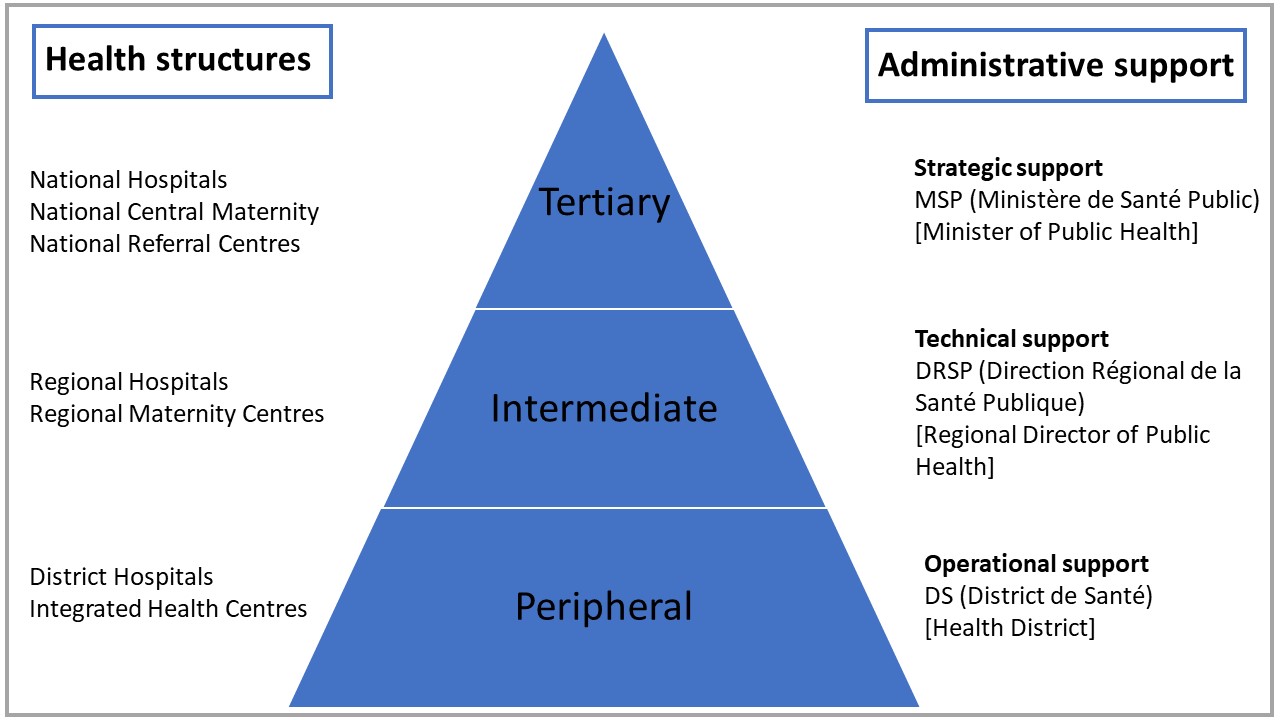
**

***Supplementary Table* *2*:** *Number of encounters that received at least one antibiotic by patients’ age, by study site and by out-patients (OP)/In-patients (IP), Uganda*

|  | **MCC** | **HICH** | | **KHC** | | **MRRH** | | **Total** |
| --- | --- | --- | --- | --- | --- | --- | --- | --- |
|  | **OP** | **IP** | **OP** | **IP** | **OP** | **IP** | **OP** |  |
| **All participants** |  |  |  |  |  |  |  |  |
| Number of encounters, n | 308 | 238 | 1995 | 35 | 417 | 309 | 563 | 3865 |
| Encounters with antibiotics prescribed, n | 154 | 115 | 982 | 8 | 274 | 138 | 168 | 1839 |
| % encounters with antibiotics prescribed | 50.0 | 48.3 | 49.2 | 22.9 | 65.7 | 44.7 | 29.8 | 47.6 |
| **Participants <5 years-old** |  |  |  |  |  |  |  |  |
| Number of encounters, n | 143 | 202 | 1504 | 25 | 194 | 267 | 438 | 2773 |
| Encounters with antibiotics prescribed, n | 65 | 94 | 709 | 8 | 127 | 124 | 142 | 1269 |
| % Encounters with antibiotics prescribed | 45.5 | 46.5 | 47.1 | 32.0 | 65.5 | 46.4 | 32.4 | 45.8 |
| **Participants 5-17 years-old** |  |  |  |  |  |  |  |  |
| Number of encounters, n | 165 | 36 | 491 | 10 | 223 | 42 | 125 | 1092 |
| Encounters with antibiotics prescribed, n | 89 | 21 | 273 | 0 | 147 | 14 | 26 | 570 |
| % Encounters with antibiotics prescribed | 53.9 | 58.3 | 55.6 | 0.0 | 65.9 | 33.3 | 20.8 | 52.2 |

MCC= Mbarara Municipal City Council Health Centre III; HICH=Holy Innocent Children’s Hospital; KHC=Kabwohe Health Centre IV; MRRH=Mbarara Regional Referral Hospital; OP=Out-patient; IP=In-patient

***Supplementary Table 3a.*** *Description of participants that received at least one antibiotic and antibiotics prescription by study site and by patient type in Niger*

| **Health structure** | **HNN** | **CHR** | **HD** | **CSI** |
| --- | --- | --- | --- | --- |
|  | **In-patients** | **In-patients** | **In-patients** | **Out-patients** |
| Total participants (n) | 124 | 164 | 190 | 182 |
| Female, n (%) | 56 (45.2) | 71 (43.3) | 80 (42.1) | 79 (43.4) |
| **Age of participants** |  |  |  |  |
| Mean age, years (SD) | 3.31 (3.92) | 2.77 (3.93) | 1.15 (2.44) | 2.94 (4.97) |
| Total <5 years, n (%) | 85 (68.5) | 118 (72.0) | 162 (85.3) | 136 (74.1) |
| Total ≥5 years, n (%) | 39 (31.5) | 46 (28.0) | 28 (14.7) | 46 (25.3) |
| Mean age children <5 years, years (SD) | 1.60 (1.33) | 1.88 (1.36) | 2.00 (1.34) | 1.85 (1.24) |
| Mean age children ≥5 years, years (SD) | 8.23 (2.92) | 8.30 (2.94) | 6.46 (2.35) | 10.78 (3.77) |
| **Antibiotic prescription** |  |  |  |  |
| Total antibiotics treatment (n) | 230 | 363 | 339 | 226 |
| Total injectable antibiotics (%) | 218 (94.8) | 309 (85.1) | 246 (72.6) | 15^1^ (6.6) |
| Children with at least one injectable, % | 100 | 98.2 | 95.8 | 8.2^1^ |
| Mean duration, days (SD) | 5.22 (2.19) | 3.55 (1.90) | 3.50 (1.15) | 4.77 (1.23) |
| Injectable | 5.27 (2.19) | 3.45 (1.92) | 3.04 (0.83) | 1 (0)^1^ |
| Oral | 3.5 (1.52) | 4.87 (0.87) | 4.89 (0.84) | 5.07 (0.63) |
| Children with multiple different antibiotics^2^, n (%) | 87 (70.2) | 150 (91.5) | 103 (54.2) | 43 (23.6) |
| Average of antibiotics per child (SD) | 1.85 (0.72) | 2.21 (0.59) | 1.78 (0.84) | 1.24 (0.44) |
| Average of treatment lines per child^3^ (SD) | 1.32 (0.69) | 1.45 (0.63) | 1.47 (0.57) | 1.07 (0.28) |
| Children with combination antibiotics^4^, n (%) | 68 (54.8) | 120 (73.1) | 61 (32.1) | 31 (17.0) |
| **Most common antibiotics prescribed, n (%)** |  |  |  |  |
|  | Ceftriaxone  85 (37.0) | Ceftriaxone+ Gentamicin  105 (28.9) | Amoxicillin  145 (42.8) | Amoxicillin  111 (49.1) |
|  | Ceftriaxone+ Gentamicin  51 (22.2) | Gentamicin  44 (12.1) | Ceftriaxone  87 (25.7) | Cotrimoxazole  21 (9.3) |
|  | Gentamicin  40 (17.4) | Amoxicillin-Clavulanic acid  42 (11.6) | Ampicillin  64 (18.9) | Ampicillin  16 (7.1) |

CSI=Centre de Santé Intégré de Madarounfa; HD=district hospital of Guidan-Roumdji; CHR= Regional Hospital of Maradi; HNN=National Hospital of Niamey.

^1^ one dose of injectable antibiotic for severe cases before referral. No hospitalized patients.

^2^ Multiple antibiotics: More than one antibiotic prescribed during treatment. This includes changes of antibiotic regimens during the course of treatment.

^3^ Treatment line: An occurrence of an antibiotic prescription. Where more than 1 antibiotic was prescribed at the same time (combination), this was considered as one treatment line.

^4^ Combination antibiotics: More than one antibiotic prescribed at the same time e.g., Ceftriaxone + Gentamicin

***Supplementary Table 3b.*** *Description of participants that received at least one antibiotic and antibiotics prescription by study site and by patient type in Uganda*

|  | **MCC** | **HICH** | | **KHC** | | **MRRH** | |
| --- | --- | --- | --- | --- | --- | --- | --- |
|  | **Out-patients** | **In-patients** | **Out-patients** | **In-patients** | **Out-patients** | **In-patients** | **Out-patients** |
| Total participants (n) | 153 | 122 | 855 | 8 | 270 | 137 | 77 |
| Female, n (%) | 68 (44.4) | 72 (59.0) | 422 (49.4) | 6 (75) | 115 (42.6) | 62 (45.2) | 48 (62.3) |
| **Age of participants** |  |  |  |  |  |  |  |
| Mean age, years (SD) | 7.89 (6.12) | 2.47 (2.95) | 3.61 (3.15) | 1.43 (0.78) | 7.19 (5.53) | 1.23 (2.80) | 3.97 (3.46) |
| Total <5 years, n (%) | 65 (42.5) | 100 (82.0) | 607 (71.0) | 8 (100.0) | 124 (45.9) | 123 (89.8) | 49 (63.6) |
| Total ≥5 years, n (%) | 88 (57.5) | 22 (18.0) | 248 (29.0) | 0 | 146 (54.1) | 14 (10.2) | 28 (36.4) |
| Mean age children <5 years, years (SD) | 1.97 (1.27) | 1.30 (1.11) | 1.91 (1.18) | 1.43 (0.78) | 2.06 (1.31) | 0.37 (0.78) | 1.83 (1.20) |
| Mean age children ≥ 5 years, years (SD) | 12.27 (4.32) | 7.80 (2.85) | 7.78 (2.52) | N/A | 11.54 (3.70) | 8.80 (2.69) | 7.71 (2.89) |
| **Antibiotic prescription** |  |  |  |  |  |  |  |
| Total antibiotics treatment (n) | 162 | 226 | 1028 | 10 | 297 | 196 | 91 |
| Total injectable antibiotics (%) | 3 (1.9) | 220 (97.3) | 58 (5.6) | 8 (80.0) | 2 (0.67) | 187 (95.4) | 4 (4.4) |
| Children with at least one injectable % | 2.0 | 98.4 | 5.9 | 100.0 | 0.74 | 98.5 | 3.9 |
| Mean duration, days (SD) | 5.10 (1.89) | 3.31 (2.29) | 6.32 (3.42) | 4.11 (1.05) | 4.96 (0.95) | 4.06 (2.93) | 4.13 (0.99) |
| Injectable | 2.33 (2.31) | 3.24 (2.26) | 2.02 (2.41) | 4 (1.07) | 2 (1.41) | 4.10 (2.92) | 3.5 (0.71) |
| Oral | 4.97 (0.62) | 5.8 (1.79) | 6.34 (3.07) | 5 (NA) | 4.97 (0.88) | 0.5 (0.71) | 4.33 (1.03) |
| Topical | 10.4 (8.85) | 7 (NA) | 9.13 (4.41) | -- | 5.3 (1.77) | -- | -- |
| Children with multiple different antibiotics^1^, n (%) | 9 (5.9) | 92 (75.4) | 155 (18.1) | 1 (12.5) | 25 (9.3) | 51 (37.2) | 13 (16.9) |
| Average of antibiotics prescribed per child (SD) | 1.06 (0.24) | 1.85 (0.58) | 1.20 (0.45) | 1.25 (0.71) | 1.1 (0.32) | 1.43 (0.62) | 1.18 (0.42) |
| Average of treatment lines per child^2^ (SD) | 1 (0) | 1.02 (0.13) | 1.00 (0.03) | 1.13 (0.35) | 1 (0) | 1.08 (0.27) | 1.01 (0.11) |
| Children with combination antibiotics^3^, n (%) | 4 (2.61) | 92 (75.4) | 110 (12.9) | 0 (0) | 9 (3.33) | 38 (27.7) | 4 (5.2) |
| **Most common antibiotics prescribed, n(%)** |  |  |  |  |  |  |  |
|  | Amoxicillin  103 (67.3) | Ampicillin+ Gentamicin  33 (26.6) | Amoxicillin-Clavulanic acid  400 (46.8) | Gentamicin  3 (33.3) | Amoxicillin  173 (64.1) | Ampicillin  67 (45.3) | Cefixime 21 (26.9) |
|  | Metronidazole  8 (5.23) | Procaine benzylpenicillin+ Gentamicin  23 (18.55) | Cefixime  144 (16.86) | Ampicillin  2 (22.22) | Metronidazole  19 (7.04) | Ampicillin+ Gentamicin  20 (8.11) | Amoxicillin  18 (23.08) |
|  | Ciprofloxacin and Cotrimoxazole  7 (4.58) | Ceftriaxone+ Procaine benzylpenicillin 21 (16.94) | Amoxicillin  48 (5.62) | Ceftriaxone  2 (22.22) | Ampicillin+ Cloxacillin  16 (5.93) | Ceftriaxone 12 (8.11) | Ampicillin+ Cloxacillin  9 (11.54) |

MCC= Mbarara Municipal City Council Health Centre III; HICH=Holy Innocent Children’s Hospital; KHC=Kabwohe Health Centre IV; MRRH=Mbarara Regional Referral Hospital

^1^ Multiple antibiotics: More than one antibiotic prescribed during treatment. This includes changes of antibiotic regimens during the course of treatment.

^2^ Treatment line: An occurrence of an antibiotic prescription. Where more than 1 antibiotic was prescribed at the same time (combination), this was considered as one treatment line.

^3^ Combination antibiotics: More than one antibiotic prescribed at the same time e.g., Ampicillin + Gentamicin.
